# Exploring the pathological mechanisms underlying Cohen syndrome

**DOI:** 10.3389/fnins.2024.1431400

**Published:** 2024-07-01

**Authors:** Fabrizio Vacca, Binnaz Yalcin, Muhammad Ansar

**Affiliations:** ^1^Department of Ophthalmology, University of Lausanne, Jules Gonin Eye Hospital, Fondation Asile Des Aveugles, Lausanne, Switzerland; ^2^Inserm UMR1231, Université de Bourgogne, Dijon, France; ^3^Advanced Molecular Genetics and Genomics Disease Research and Treatment Centre, Dow University of Health Sciences, Karachi, Pakistan

**Keywords:** VPS13B, COH1, BLTP, Golgi, neurodevelopment, membrane contact sites, lipid transfer protein

## Abstract

Cohen Syndrome (CS) is a rare autosomal recessive disorder caused by biallelic mutations in the *VPS13B* gene. It is characterized by multiple clinical features, including acquired microcephaly, developmental delay, intellectual disability, neutropenia, and retinal degeneration. VPS13B is part of the bridge-like lipid transport (BLTP) protein family, which in mammals also includes VPS13A, -C, and -D. The proteins of this family are peripheral membrane proteins with different sub-cellular localization, but all share similar structural features and have been proposed to act as lipid transport proteins at organellar membrane contact sites. VPS13B is localized at the Golgi apparatus and is essential for the maintenance of organelle architecture. Here we present a review of the experimental data on the function of the protein at the cellular level, discussing the potential link with disease phenotype and review the studies on animal models recapitulating features of the human disease.

## Introduction

Cohen Syndrome (CS) (MIM: 216550) is a multisystemic genetic disease which was initially described five decades ago (Cohen et al., 1973). It is caused by biallelic mutations in the *VPS13B* gene. Over 660 pathogenic variants have been identified so far, mostly truncating loss-of-function (LoF) mutations.^1^ It affects an estimated 50000 individuals worldwide, projected based on the prevalence of predicted LoF variants in *VPS13B* in the gnomAD database (Chen et al., 2024). Patients with CS suffer from many symptoms including developmental delay, intellectual disability, post-natal microcephaly, metabolic issues, neutropenia, severe myopia, and progressive loss of vision due to retinal degeneration (Kivitie-Kallio et al., 2000; Kivitie-Kallio and Norio, 2001). Brain structural abnormalities have been reported in some patients using cerebral magnetic resonance imaging (MRI). These include thick corpus callosum (Kivitie-Kallio et al., 1998; Mochida et al., 2004; Rejeb et al., 2017; Koehler et al., 2020), thin corpus callosum (Alipour et al., 2020), atrophy of the cerebellar vermis (Mochida et al., 2004; Waite et al., 2010), pontocerebellar atrophy (Katzaki et al., 2007), thinning of the cortex (Hu et al., 2021) and cerebral atrophy (Ghzawi et al., 2021). The disease-causing gene was identified in 2003 and originally named *COH1*, later renamed *VPS13B*, by homology with the yeast protein (Kolehmainen et al., 2003). In this mini-review, we outline the current experimental data that show potential pathological mechanisms of CS. This encompasses various models, including cell, neuronal, and animal models.

## Cellular functions of VPS13B protein

**Figure 1:**
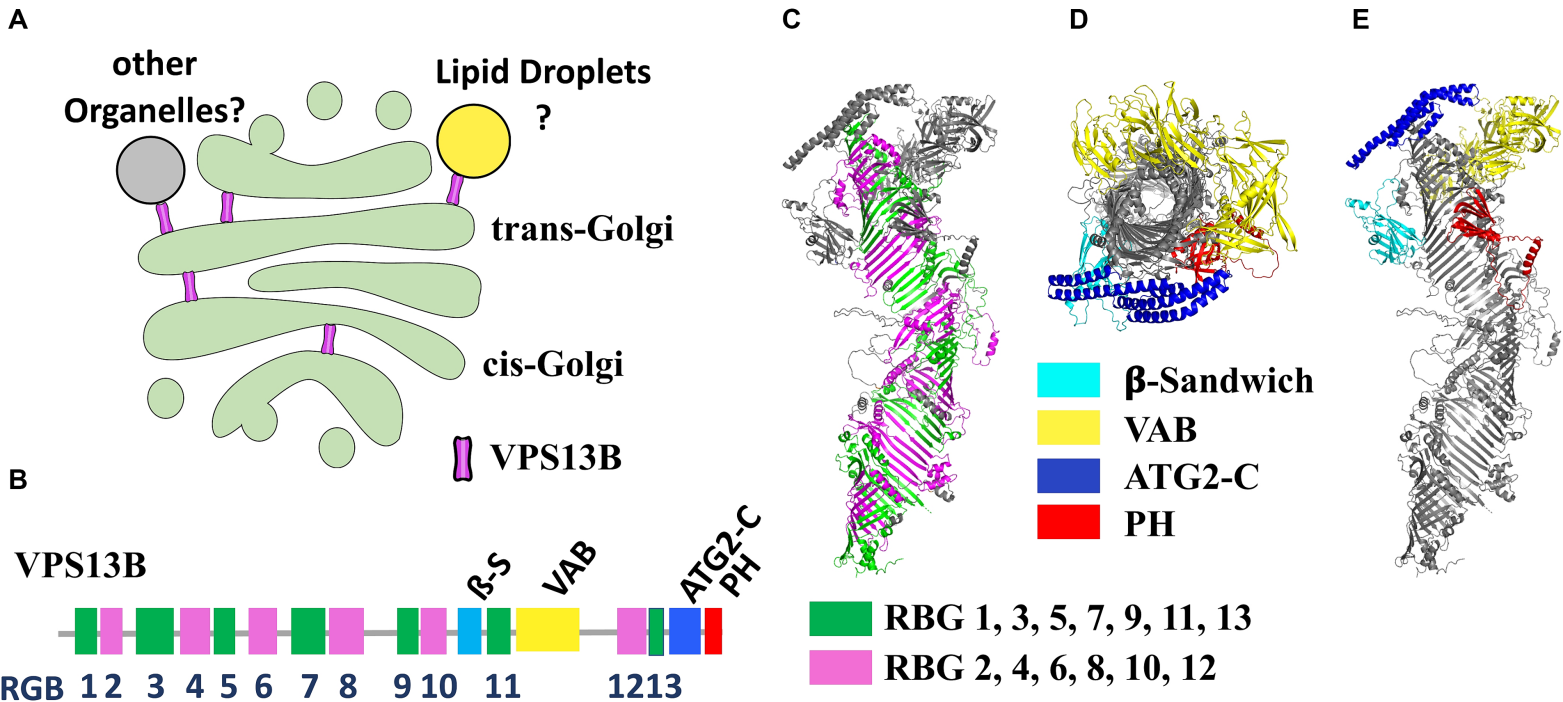
**(A)** Illustration of the potential localization of VPS13B within the Golgi complex according to available experimental evidences. **(B)** Scheme of the domain organization of the VPS13B polypeptide highlighting the position of the RBG repeats and the folded domains. **(C)** Side view of the predicted alphafold structure of VPS13B highlighting the RBG repeats forming the central scaffold of the protein (alternate RBG colored in green and magenta). RBG boundaries have been designed according to Levine (Levine, 2022). **(D,E)** Top view (from C-terminus) **(D)** of the same structure which clearly shows the tunnel going through the center of the protein and side view **(E)** highlighting the positions of the four folded domains. VPS13B Alphafold structure prediction was deposited by Samander et al. (DOI: 10.5281/zenodo.7626581).

*VPS13B* is one of the four mammalian genes (along with *VPS13A, -C, and -D*), homologous of the yeast *VPS13* (Velayos-Baeza et al., 2004). Of the four mammalian *VPS13* genes, *VPS13B* is the more evolutionary distant, proposed to have split very early from the other three in eukaryotic evolution. Recent clustering analyses suggest that the last eukaryotic common ancestor likely contained two *VPS13* paralogs: ancestral forms of *VPS13A/C/D* and *VPS13B* (Levine, 2022). These proteins are now recognized as part of a larger protein family denominated bridge-like lipid transfer protein (BLTP) which also includes the autophagy factor ATG2 and other few proteins (Neuman et al., 2022; Hanna et al., 2023). A remarkable structural feature of this protein family is the organization into a series of repeated units known as RBG (repeated beta-groove) units that compose the core of the hydrophobic tunnel and constitute the central backbone of the structure (Levine, 2022). The N- and C-terminal regions are the most conserved across different isoforms and contain accessory domains that likely play a crucial role in membrane interaction and lipid mobilization. VPS13B contains four folded accessory domains in the C-terminal region protruding from the rod-like structure formed by the RBG domains (Figure 1). Three of these domains are common across all VPS13 proteins: the arc-shaped Vps13 adaptor binding (VAB) domain, which is pivotal for protein–protein interactions; the ATC2-C domain, also known as the Gondola domain, composed of four amphipathic α-helices, also present in the lipid droplet proteins PLIN2 and GPAT4 and likely mediating interaction with lipid bilayers; a pleckstrin homology (PH) domain, which facilitates phosphoinositide binding. Additionally, the VPS13B protein uniquely contains a jellyroll/β-sandwich domain with an unclear function (Levine, 2022; Dall'Armellina et al., 2023; Hanna et al., 2023). BLTP proteins have been proposed to transfer lipids through a hydrophobic tunnel between adjacent organelle membranes at membrane-contact sites (MCS). This hypothesis is supported by experimental data from structural analyses, electron microscopy and biochemical studies (Kumar et al., 2018; Li et al., 2020; Cai et al., 2022). Evidence from specific cases, such as ATG2 in autophagosome expansion (Valverde et al., 2019) and yeast Vps13p in prospore formation, (Nakamura et al., 2021), suggests that these proteins contribute to membrane expansion by bulk lipid flow. However, no lipid selectivity has been definitively demonstrated to date. BLTP proteins are thought to connect two organelles with their N- and -C terminal domains, theoretically enabling them to serve also as scaffolding proteins at MCS.

VPS13A, -C, and -D are generally assumed to connect the endoplasmic reticulum (ER) to other organelles, as detailed by Hanna et al. (2023). However, no evidence currently exists to show that VPS13B connects to the ER. This is consistent with the lack of a FFAT motif in the N-terminal region, a motif that mediates ER interaction in other VPS13 proteins. Several reports show instead that VPS13B predominantly localizes to the Golgi complex. Interestingly, silencing RAB6, a VPS13B interactor, was found to partially prevent localization of VPS13B to the Golgi, highlighting its potential role in Golgi association (Seifert et al., 2015). Although it remains unclear whether VPS13B establishes connections between the Golgi and other organelles, immunofluorescence after hypotonic-mediated organelle swelling, indicates that VPS13B may localize at the interface between cis- and trans-Golgi membranes, potentially forming MCS between different Golgi cisternae (Ugur et al., 2023). VPS13B has also been found in association with lipid droplets, particularly in a subset of lipid droplets in close contact with the Golgi (Du et al., 2023; Figure 1). While localization in the Golgi complex seems well established and was observed with endogenous protein staining (Seifert et al., 2011, 2015; Zorn et al., 2022), other data mostly rely on over-expressed proteins, due to intrinsic technical challenges of visualizing the endogenous protein with available antibodies. Therefore, these localization findings should be interpreted cautiously.

Up to 100 VPS13B potential binding partners have been identified in high-throughput protein interaction screenings.^2^ Some of these interactions have been validated through targeted experiments: for instance, RAB6, a GTPase which plays a prominent role in the regulation Golgi traffic (Seifert et al., 2015); and the SNARE proteins Syntaxin 6 (STX6) and Syntaxin 13 (STX13) (Koike and Jahn, 2019). STX6 is particularly notable due to its involvement in multiple vesicular trafficking pathways, including RAB6-dependent retrograde traffic from endosomes to the Golgi (Mallard et al., 2002; Ganley et al., 2008). Curiously, VPS13B was reported to bind selectively to vesicles positive for both STX6 and STX13 and is implicated in trafficking between early and recycling endosomes rather than the Golgi (Koike and Jahn, 2019). Moreover, Family With Sequence Similarity 177 Member A (FAM177A), a Golgi-localized protein of unknown function, has been proposed as a functional interactor of VPS13B. FAM177A is linked to a neurodevelopmental genetic disorder with phenotypic similarities to CS and exhibits a cellular phenotype similar to that of VPS13B (Ugur et al., 2023).

## Golgi fragmentation as the central cellular defect in Cohen syndrome

Golgi fragmentation is a prominent and characteristic cellular defect observed in VPS13B-depleted cells (Seifert et al., 2011). A functional impairment of the Golgi is further supported by protein glycosylation defects in serum samples from CS patients (Duplomb et al., 2014). Although the correlation between VPS13B localization and Golgi fragmentation is strong, the exact role of VPS13B in this phenomenon as well as its broader role in maintaining Golgi morphology remains unclear. A recent study investigating the ability of missense variants to restore Golgi morphology further reinforces the association between Golgi fragmentation and CS pathogenicity (Zorn et al., 2022). Additionally, genome-wide CRISPR screening identified VPS13B among the top genes required for cholera toxin mediated intoxication, alongside other Golgi proteins (Gilbert et al., 2014). This finding implies a potential role in Golgi retrograde trafficking, although establishing a cause-and-effect relationship would require further investigations. The observation of Ugur et al. (2023) of a potential cis-to-trans Golgi bridging function of VPS13B aligns with this hypothesis. So far, the better-established inter-Golgi retrograde traffic mechanism is vesicle-mediated and is orchestrated by the Conserved Oligomeric Golgi (COG) complex in cooperation of set of Golgi SNARE proteins. Interestingly, the deletion of COG components often leads to Golgi fragmentation. Even if experimental evidences will be necessarily required to address VPS13B mechanistic function within the Golgi, we could speculate that it could be essential in facilitating retrograde traffic of even directly mediate the retrograde flow of some lipid species among the cisternae of the organelle.

Regardless of whether VPS13B directly causes Golgi fragmentation, this phenotype is likely linked to CS pathology. Although it is challenging to directly correlate Golgi impairment to neurodevelopmental features of CS because the Golgi apparatus is involved in many cellular processes, certain observations suggest a causal link. Similarities have been observed between CS and other diseases that affect Golgi morphology and trafficking, collectively known as “Golgipathies” (Passemard et al., 2017). These diseases include disorders impacting Golgi-associated retrograde protein (GARP) complex-mediated endosome-to-Golgi retrograde traffic (Feinstein et al., 2014; Gershlick et al., 2019); coating proteins involved in Golgi-to-ER traffic via COPI vesicles (Macken et al., 2021; Marom et al., 2021); Golgins like GM130 (Shamseldin et al., 2016), and tethering complexes facilitating bidirectional ER-Golgi traffic, such as Trafficking protein particle (TRAPP) (Sacher et al., 2019) and COG (D'Souza et al., 2020). Despite the heterogenicity of these conditions, they share key neurodevelopmental features with CS, such as postnatal microcephaly, often accompanied by dysgenesis of the corpus callosum. Unlike primary microcephaly, which mostly affects neurogenesis and migration during embryonic development, secondary (postnatal) microcephaly likely results from later events such as neuronal maturation, synaptogenesis and myelination.

## Neuronal models of Cohen syndrome

Recent studies on neuronal models of CS investigated the correlation of VPS13B depletion or knockdown (KD) with neurodevelopmental abnormalities. In rat primary hippocampal neurons, VPS13B KD initially led to axonal elongation defects and misalignment of the Golgi complex with the direction of elongation, suggesting a correlation between Golgi defects and the neurodevelopmental phenotype (Seifert et al., 2015). Further investigations using human induced pluripotent stem cell (iPSC)-derived neurons have provided a valuable model for studying the neurodevelopmental features of CS. Lee et al. reported that neurons derived from a patient-derived iPSC normally differentiate into glutamatergic neurons but exhibit defective synaptogenesis and reduced expression of synaptic genes. Additionally, neurospheres generated from the same iPSC showed reduced size, potentially recapitulating microcephaly in CS patients (Lee et al., 2020a). The same laboratory also observed an increased autophagic flux both in fibroblasts and differentiated neurons (Lee et al., 2020b). In another study, iPSC-derived neurons from two CS patients using an alternative differentiation protocol revealed ultrastructural defects in several organelles beyond the Golgi, including altered morphology of ER and mitochondria, as well as increased MCS between these organelles. An impairment in autophagic flux was also noted, which aligns with elevated cellular stress and potential neurodegeneration (Shnaider et al., 2023). Given the well-known heterogenicity of iPSC lines, these studies (each using fibroblasts from only one or two patients) require further validation to fully establish how well they recapitulate the pathological mechanism of the disease at the cellular level. Nevertheless, *in vitro* neuronal cultures as well as brain or retinal organoids, that will be hopefully established in a near future, represent promising systems for investigating the cellular pathology of CS and exploring potential therapeutic targets.

## Animal models of Cohen syndrome

CS has been also investigated in animal models over the past decade. These models recapitulate many of the features seen in human patients, offering an invaluable tool to explore the disease’s pathological mechanisms and identify potential therapeutic strategies. An overview of model organisms used in CS research, along with their associated phenotypic traits, is summarized in Table 1.

**Table 1:** Summary table of phenotypic characteristics linked to animal models of Cohen Syndrome.

Animal models of Cohen syndrome	Dog	Mouse	Mouse	Mouse			
Nomenclature/breed	Border Collies	*Vps13b* < em1(IMPC)Tcp>	*Vps13b* < em1Yosl>	Vps13b < tm1.2Ics>			
Genetic background	Not applicable	C57BL/6 NCrl	C57BL/6 N	Mixed C57BL/6 N-C57BL/6 J			
Sample size (control animals)	10 obligate carriers	419 M and 430 F	9 M/F combined	12 M	3 (sex unknown)	12 M and 9 F	16 M and 16 F
Sample size (mutant animals)	7 dogs (sex unknown)	8 M and 8 F	12 M/F combined	12 M	3 (sex unknown)	11 M and 5 F	16 M and 16 F
Phenotypes							
- Lethality	+	−	NA	NA	NA	NA	+
- Developmental delay	+	+	−	NA	NA	NA	+
- Behavior							
Intellectual disability	+	NA	+	NA	NA	NA	+
Abnormal social behaviors	NA	NA	−	NA	NA	NA	+
Abnormal anxiety-like behaviors	NA	NA	−	NA	NA	NA	+
- Abnormal locomotor activity	NA	+	+	NA	NA	NA	+
- Impaired motor coordination	NA	NA	+	NA	NA	NA	+
- Hypotonia	+	NA	+	NA	NA	NA	+
- Brain anatomy							
Postnatal microcephaly	+	NA	−	NA	NA	NA	+
Hippocampal hypoplasia	NA	NA	NA	NA	NA	NA	+
Other neuroanatomical phenotypes	NA	NA	NA	NA	NA	NA	+
- Eye							
Cataracts	NA	+	−	NA	+	NA	NA
Abnormal retina morphology	NA	+	−	NA	+	NA	NA
- Face							
High narrow palate	+	NA	NA	NA	NA	+	NA
Prominent nasal bridge	+	NA	NA	NA	NA	+	NA
- Skull shape anomalies	NA	NA	NA	NA	NA	NA	+
- Reproduction							
Male infertility	NA	+	NA	+	NA	NA	NA
Delayed puberty	+	NA	NA	NA	NA	NA	NA
- Short stature	+	+	−	NA	NA	NA	+
- Truncal obesity	−	−	NA	NA	NA	NA	NA
- Neutropenia	+	NA	NA	NA	NA	NA	NA
References	Shearman and Wilton (2011)	https://www.mousephenotype.org/data/genes/MGI:1916380	Kim et al. (2019)	Da Costa et al. (2020)	Lhussiez et al. (2020)	Bonfante et al. (2021)	Montillot et al. (2023)

NA, Not Available; Plus sign, presence; Minus sign, absence; em1, endonuclease-mediated mutation 1; IMPC, International Mouse Phenotyping Consortium; Tcp, Toronto Centre for Phenogenomics; Yosl, Yong-Seok Lee; tm1.2, targeted mutation 1.2; Ics, Mouse Clinical Institute; M, Male; F, Female.

The first animal model for CS was established in 2011, with a naturally occurring *VPS13B* variant identified in Border Collie dogs, displaying trapped neutrophil syndrome. Linkage analysis on a pedigree of dogs traced back to a single common ancestor and a candidate gene approach led to the identification of a recessive 4-bp deletion in exon 19 of the largest transcript of VPS13B, which results in premature truncation of the protein (Shearman and Wilton, 2011). Seven dogs harboring the deletion in the VPS13B gene exhibited phenotypic traits similar to those of human CS patients. With the exception of the truncal obesity absent in the pedigree and ophthalmic features, not examined in this study, these dogs showed the main characteristics of CS patients including developmental delay, intellectual disability, postnatal microcephaly, facial abnormalities (short philtrum, high narrow palate, prominent nasal bridge and prominent central upper incisors), along with hypotonia, delayed puberty, short stature, and neutropenia (Table 1). Intellectual disability was estimated in these dogs according to their reduced ability to learn simple tasks. According to the report, 43% of the dogs (3 out of 7) displayed a defective learning phenotype. It is worth mentioning that among the seven dogs, two died before reaching maturity, and their sex was not recorded.

In recent years, three mouse models for CS have been developed, all of which are null/knockout (KO) models. The first two were generated using CRISPR/Cas9 endonuclease-mediated gene disruption, while the third employed a traditional targeted strategy (details are provided in Table 1). The first model was engineered by the Toronto Centre for Phenogenomics, partner of the International Mouse Phenotyping Consortium (IMPC), which aims to produce knockout mice for every coding gene in the mouse genome (White et al., 2013). This first allele, designated as *Vps13b*^em1(IMPC)Tcp^ was developed from a pure genetic background C57BL/6 NCrl, a 2,348-bp deletion was introduced on mouse chromosome 15, resulting in the deletion of exons 2–4 of *Vps13b* gene. Phenotypic data of this model are available to the scientific community through the IMPC website.^3^ Phenotypic data of 16 KO mice (*n* = 8 per sex) revealed decreased exploration in new environment, reduced body length, abnormal lens and retina morphology, and male infertility (summarized in Table 1).

The second CS model, *Vps13b*^em1Yosl^, was developed in the laboratory of Yong-Seok Lee at the Seoul National University College of Medicine. This model was engineered on a pure C57BL/6 N genetic background by deleting exon 2 of *Vps13b*, specifically removing 156 bp that includes the start codon (Kim et al., 2019). Phenotypic data from male and female KO mice were combined (*n* = 12 KO mice). These mice exhibited significant memory and cognitive impairments in the Morris water maze paradigm, reduced activity in the open field test, and decreased motor coordination in the rotarod test, evidenced by a shorter latency to fall. Intriguingly, the study indicated normal anxiety-like and social behaviors in this model. Importantly, no morphological changes, eye abnormalities, or developmental delays were observed in these mice (see Table 1).

The third model, denoted as *Vps13b*^tm1.2Ics^, is the most extensively characterized model of CS. This model involved the deletion of exon 4 in the *Vps13b* gene (Da Costa et al., 2020; Lhussiez et al., 2020; Bonfante et al., 2021; Montillot et al., 2023). Unlike the two previous mouse models, it was developed on a mixed genetic background comprising C57BL/6N and C57BL/6J strains of mice, which are known to have genetic and phenotypic divergences (Simon et al., 2013). This model presented male infertility (Da Costa et al., 2020) and severe forms of cataracts (Lhussiez et al., 2020). In cranio-skeletal analysis using Skyscan 1174 micro-computed tomography, *Vps13b*^tm1.2Ics^ mice displayed craniofacial abnormalities, mainly affecting the palate (narrowing and elevation) and the nasal region (elongation and widening of the nasal tip). These abnormalities were more pronounced in males than females (Bonfante et al., 2021).

Furthermore, brain anatomy and behavior were comprehensively assessed in *Vps13b*^tm1.2Ics^ mice (Montillot et al., 2023). About half of the KO mice died during the first week of life, while the remaining mice had normal lifespan and presented core phenotypes of CS, including microcephaly, growth delay, hypotonia, altered memory, and enhanced sociability. A quantitative 2D histological assessment of 40 morphological parameters across 22 different brain regions (Mikhaleva et al., 2016; Collins et al., 2018; Nguyen et al., 2022) revealed that the hippocampus, crucial for memory and spatial orientation, was one of the most affected regions, with its volume reduced by around 40%. While both sexes were affected, certain neuroanatomical and behavioral phenotypes were less pronounced in females.

## Discussion

In this review, we summarize two decades of experimental research on VPS13B and CS, spanning from basic cell biology to the more recent work on mouse models. The efforts in characterizing the mechanistic functions of VPS13B, along with the research on other members of the BLTP protein family, significantly enhanced our understanding of the basic functions of these proteins. Recent studies suggest a role in intra-Golgi traffic whose impairment leads to a partial disruption of the morphology of the organelle. The similarity of CS with other disorders caused by mutations in Golgi complex proteins suggests that Golgi dysfunction may play a role in disease etiology. However, many questions remain open about the precise function of VPS13B, including its lipid selectivity and its potential role beyond lipid transport as a scaffold protein or tethering factor at membrane contact sites. Addressing these questions is essential for developing a comprehensive understanding of the pathological mechanisms of CS.

Modeling the disease in physiologically relevant cellular models will also be crucial. Efforts have been made to investigate the role of VPS13B in neuronal development using iPSC-derived neuronal cells. While these findings are still preliminary and do not yet establish a direct correlation between observed cellular phenotypes and the *in vivo* features of the disease, they represent a promising approach for understanding the disease mechanisms at the cellular level.

The studies on mouse models have demonstrated reproducibility and high clinical relevance, mirroring several features of CS patients, such as developmental delay, secondary microcephaly, craniofacial gestalt, hypotonia, and positive-friendly behaviors. Importantly, mouse studies revealed previously unreported neuroanatomical features, including hippocampal and cerebellar atrophy attributed to early-life neuronal loss, which could be used as robust readouts of the disease. While both sexes were affected, mouse and human data suggest that VPS13B plays a more prominent role in males than females. Hence, it is crucial to consider sex as a biological variable in future pre-clinical studies on CS.

## Author contributions

FV: Writing – original draft, Writing – review & editing. BY: Writing – original draft, Writing – review & editing. MA: Writing – original draft, Writing – review & editing.

## Footnotes

^1^www.ncbi.nlm.nih.gov/clinvar

^2^https://thebiogrid.org

^3^https://www.mousephenotype.org/
